# The detrimental impact of high environmental temperature on physiological response, growth, milk production, and reproductive efficiency of ruminants

**DOI:** 10.1007/s11250-023-03805-y

**Published:** 2023-11-01

**Authors:** Alsaied Alnaimy Habeeb, Samir F. Osman, Fatma E. I. Teama, Ahmed E. Gad

**Affiliations:** Egyptian Atomic Energy Authority, Nuclear Research Center, Radioisotopes Applications Division, Biological Applications Department, Cairo, P.O. 13759 Egypt

**Keywords:** Ruminants, Physiological response, Heat stress, Growth, Milk yield, Reproductive efficiency

## Abstract

The optimal environments for ruminants are air temperatures between 13 and 20 °C, winds between 5 and 18 km/h, humidity levels between 55 and 65%, and a moderate amount of sunlight. In tropical and subtropical regions, climate is the top factor restricting animal growth and reproductive efficiency. The digestive system, blood biochemical components, and hormones all go through a range of physiological changes at high temperatures. Ruminant animals respond to heat stress by drinking more water, breathing more quickly, panting, and raising their rectal temperatures while reducing their activity levels, intake of roughage, and rumination. Blood metabolites and biochemical modifications are negatively impacted by the concentration of blood biochemical components and hormonal levels, particularly those of anabolic hormones, which are decreased as a result of the animals’ exposure to high environmental temperatures. Changes in blood metabolite and hormone levels were influenced by the duration of exposure to high temperatures, the level of background heat, and the species, breed, and age of the animals. The major biological changes caused by heat stress have a negative impact on growth, milk production, and reproduction. Animals subjected to high environmental temperatures also undergo reductions in feed intake and feed efficiency. These changes eventually impair ruminant reproduction and production abilities.

## Introduction

The World Meteorological Organization and the United Nations Environmental Program predict that, as a result of global warming, animals will be exposed to higher temperatures more frequently and for longer periods of time overall, especially during the summer season. Everywhere in the world, common hyperthermia can occasionally occur due to the prolonged sun exposure and sweltering heat of the summer season. To protect animal functions from high temperatures and to speed retrieval from hypothermic injuries, animals may undergo a number of physiological and biochemical changes (McMichael et al. 1996). Heat stress is a physiological condition that occurs when a species’ core body temperature rises above the range that is recommended for normal activity as a result of a total heat load (internal production plus environment) that is greater than the capacity for heat dissipation. This leads to physiological and behavioral reactions to lessen the strain (Bernabucci et al. 2010). Ruminants suffer significantly in terms of reproduction and productivity when exposed to extreme environmental stress during the summer season for more than 6 months each year (Habeeb et al. 1992). When animals are under heat stress, their biological processes are profoundly altered. These changes include decreased feed consumption as well as enzymatic activities and hormonal levels. These changes reduce the animals’ normal immunity, which makes them more vulnerable to infection (Habeeb et al. 2008). Ruminants, like other homoeothermic animals, decrease heat production and increase heat loss pathways during times of heat stress in an effort to maintain euthermia. Increased respiration rates, decreased feed intake, and increased water intake are the primary reactions to heat load. Because higher-producing animals produce more heat throughout their metabolic processes, they are more susceptible to heat stress (Bernabucci et al. 2010). At temperatures higher than 25 °C, animals get heat exhaustion. Deep body temperatures rise, manufacturing output declines, and animal cells are affected, and the effect is amplified when the humidity level exceeds 50%. The major biological changes caused by heat stress impair development, milk supply, and reproduction, and the disruption of the physiological equilibrium has a detrimental effect on the animals’ productivity (Kamal and Habeeb 1999). When an animal’s body temperature rises and animals are unable to release enough heat to maintain thermal balance, animals experience heat stress. Heat stress, according to Marai and Habeeb (2010), is a condition brought on by exposure to high temperatures and the physiological responses used by animals to maintain their thermal balance. Animals will consume less feed as a defensive precaution so as to generate less metabolic heat. The rate of breathing rises, and there is a great proliferation in the amount of heat that is lost insensibly through water evaporation in the lungs (Habeeb et al. 2018a). Additionally, compared to animals living in temperate climates, farm animals pee more often and lose significantly more mineral ions in their urine (Habeeb et al. 1992). Ruminants regulate their body temperature by balancing heat loss and absorption, a process known as thermoregulation. The temperature range between the lower and higher critical temperatures is known as the thermo neutrality zone. Maximum productivity and the lowest physiological cost are typically attained in this zone. The capacity of ruminants to produce meat and milk declines when the critical temperature is exceeded. Ruminants use a variety of strategies to keep their homeostasis in balance. The thermoneutral range is the range of environmental circumstances in which an animal can control heat loss with the least amount of thermal effort (Igono et al. 1985). The rates of heat generation and dissipation, as well as metabolism, are affected by changes in the ambient temperature. When the ambient temperature falls below the lower serious temperature, animal metabolism increases to produce more heat. Food intake is restricted, and evaporative heat loss rises when the ambient temperature is above the evaporative serious temperature, which reduces metabolism and heat generation (Mount 1974). Ruminants can expel body heat in a variety of ways, including through convection, radiation, and evaporation, which occur when moisture is removed from the cow’s skin via sweating and from the cow’s lungs via panting. These processes are carried out to make the animal body sheds more heat when the environmental temperature becomes extremely hot. According to Guyton (1969), warming activates the pre-optic thermostatic region and increases the animal’s heat loss by three separate mechanisms: evaporative heat loss from the skin by activating sweat glands, vasodilator nerves to the skin to accelerate the transport of heat by the blood to the body surface, and inhibiting a sympathetic center in the posterior hypothalamus, which frees the skin’s natural vasoconstrictor tone and promotes higher vasodilation. According to estimates, tropical and subtropical regions produce more than 50% of the world’s meat and 60% of its milk. With the 1990s and 2000s contributing to a rise of 0.28 °C each decade, the earth’s climate has warmed during the past century (0.74 to 0.188 °C (McMichael et al. 1996; Habeeb 2020e). According to the ICTP (2001), the rise in average global temperature by 2100 may range from 1.88 to 4.08 °C. These forecasts state that as the quantity of accessible undeveloped land decreases and the world population and food supply continue to expand quickly, particularly in tropical and subtropical countries, the detrimental effects of heat stress on cattle performance will become more and more visible.

This review article explains the impact of high environmental temperature on physiological and blood biochemical parameters, growth performance, milk yield and composition, and reproductive efficiency in farm animals.

## The negative effect of high environmental temperatures on physiological and blood biochemical responses of ruminant animals

Generally, farm animals’ responses to high environmental temperatures include increased body temperature, increased respiratory rate, panting, decreased activity, decreased feed intake (> 10% or more), decreased milk production (10–20% or more), decreased daily body gain (20–40% or more), increased sweating and marginal blood flow, decreased fertility levels, and increased mortality (Wiersma 1994). The response to heat load and the heat-induced change in home reactive modifiers impact post-absorptive energy, lipid, and protein metabolism; impair liver function; promote oxidative stress; harm the immune system; and reduce reproductive ability (Bernabucci et al. 2010). Ruminants, including the most popular cow breeds in the world, are affected by heat stress, which lowers feed consumption, milk yield, daily gain, and reproductive traits (Kadokawa et al. 2012). Due to the increased respiration, sweating, and rectal temperature under hot climate conditions, there are some irregularities in the protein, water, energy, and mineral metabolisms. Body temperature is strictly regulated by balancing heat production from the body with heat loss to the environment through conduction, convection, radiation, and evaporation (Hansen 2009).

### Feed intake

The quantity of dry matter consumed by animals in feed is decreased by high environmental temperatures. Habeeb et al. (1996) found that the dry matter intake during the hot summer season was much lower than that during the mild climate of the winter season. Ruminants living in a hot climate tend to consume more water and less dry food (Bernabucci et al. 1999). Shwartz et al. (2009) found that as environmental temperatures rose, the consumption of dry matter by nursing Holstein cows decreased noticeably. Brien et al. (2010) found that in conditions of heat stress, the body consumes roughly 12% less dry matter. Bernabucci et al. (2010) and Farooq et al. (2010) found that the initial reaction to high environmental temperatures is a reduction in food consumption. Padua et al. (1997) found that the Suffolk lambs in a climatic chamber with high temperatures (30.5 °C) dramatically decreased their daily feed intake compared to a group in a mild climate (19.3 °C). Marai et al. (2007) found that sheep subjected to heat stress consumed less feed. Similarly, as the ambient temperature rose, so did the dry matter intake per kg of live weight. Without changing the rams’ consumption of roughage, the drop in concentrate intake at 35 °C in a climate chamber was estimated to be roughly 13% (Nardone et al. 2010). The consumption of TDN, CP, and DCP was substantially higher in the winter season than in the hot summer season. All feeding values (TDN and DCP) and nutrient digestibility coefficients were dramatically reduced from the summer ration to the winter ration (Gaafar et al. 2021). The summer had substantially lower levels of DM, TDN, CP, and DCP per kg of weight gain than the winter did. The average daily gain yield, net revenue, and economic efficiency were all much greater during the winter season despite feed costs per kg of weight gain being significantly higher than they were during the hot summer season (Gaafar et al. 2021). A reduction in feed intake is the principal response to high environmental temperatures. High ambient temperatures affect the peripheral thermal sensors to transmit nerve signals to the hypothalamus and then to appetite center that suppresses the appetite, which reduces the intake of food (Habeeb et al. 1992). Additionally, because grazing, especially for ruminants, greatly contributes to the creation of body heat, animals restrict their food consumption in an effort to produce a lesser amount of metabolic heat (Kadzere et al. 2002). Reduced blood metabolite concentrations and biochemical alterations are brought on by ruminants' exposure to hot ambient temperatures (Habeeb et al. 2018c).

### Water intake

**Fig. 1:**
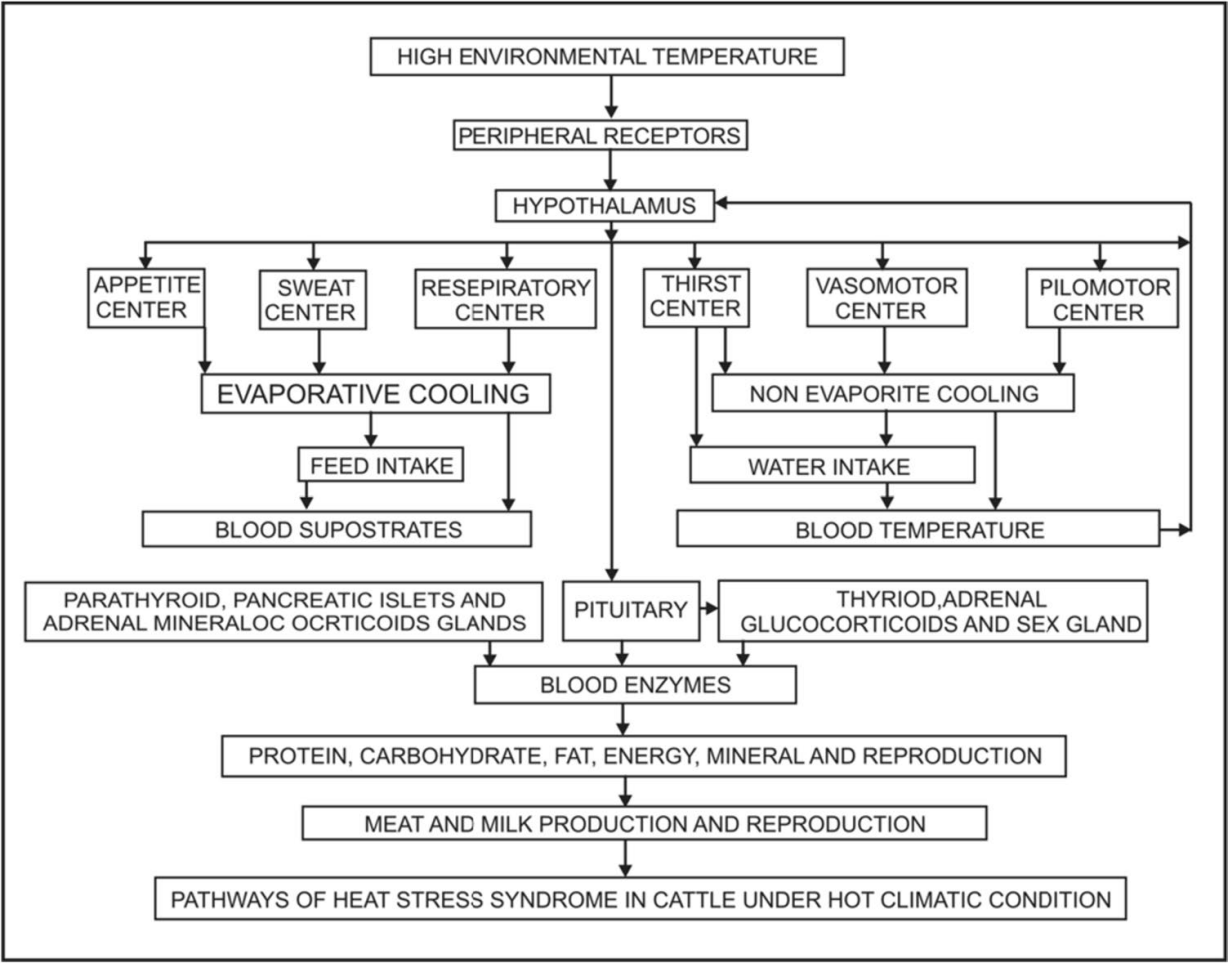
Pathways of heat stress syndrome in an animal under hot climatic conditions (Kamal 1975)

In a steady state, total water intake and total output must be equal. Ruminants drink water at least five times as much as animals do under normal temperature conditions. Animals also produce more urine and lose more mineral ions from their bodies (Bray and Bucklin 1996). The amount that must be taken depends on how much water is needed to replace total water loss and give body fluids the proper osmotic concentration. On the other hand, it is essential to provide a consistent supply to maintain a somewhat steady amount of total body water content. Since water is often freely available, the necessity for it has not been properly investigated (Habeeb et al. 2020a, 2020b). Increased water consumption in ruminant animals is associated with exposure to hotter surroundings (Brien et al. 2010). The first hazard of high environmental temperatures is quick desiccation. The calf’s body temperature is regulated through sweating and panting, and these two cooling methods need water. To replenish the lost fluid, a calf has to consume between 3 and 6 l of water each day. In the hot summer months, if this water is unavailable, the animal may quickly get dehydrated. Water consumption (L/day) significantly increased by 28.8% at THI 74.9 during the spring season and by 63.4% at greater THI (85.5) during the summer season compared to those at low THI 68.1 in winter (Habeeb et al. 2022). High ambient temperatures affect the peripheral thermal sensors to transmit nerve signals to the hypothalamus and then to thirst center that increases the water consumption as presented in Fig. 1 (Kamal 1975). This diagram explains how high ambient temperatures affect peripheral thermal sensors that transmit nerve signals to the hypothalamus and then to thirst and appetite centers that affect physiological thermoregulation, reduce the amount of dry matter consumed, increase water consumption, decrease blood biochemical metabolites as well as anabolic and sex hormones, and reduce daily body gain as well as milk yield and composition.

A deficiency of energy, substrates, and hormones may be the cause of the decreased milk output and composition. A rise in cortisol levels in ruminants exposed to high temperatures may also be related to a decrease in milk yield and quality (Habeeb et al. 2018c). In reaction to high environmental temperatures, ruminants frequently take a lesser amount of food, which of course lowers the increase in core animal body temperature caused by ingestion. The quantity of heat lost through water evaporating from the lungs during panting also increases noticeably as the frequency of breathing does.

### Blood hormones

A decrease in blood hormonal levels like T_3_, T_4_, growth hormone, insulin, and insulin-like growth factor is expected to result from exposure of the ruminants to the high environmental temperature (Habeeb et al., 2011 and 2012 ). The levels of blood cortisol, follicle-stimulating hormone, and estradiol in response to the length of heat exposure, the degree of external heat, and the species, breed, and age of the ruminant animals have resulted in contradictory results (Roth et al., 2000 and 2001). Cattle exposed to high ambient temperatures are known to have lower plasma T_3_ and T_4_ levels of thyroid hormones, such as T_4_ (Menegatos et al. 2006; Marai and Habeeb 2010; Habeeb et al. 2011). The response of T_3_ and T_4_ to heat stress is extremely gradual, according to Silanikove (2000), and it takes several days for the levels to reach a new steady state. The thyroxin hormone’s secretion can be lowered by up to 25% over time when exposed to heat stress. T_3_ is primarily related to thermogenesis and decreases gradually in heat-stressed animals, and so the low level may be a reflection of the animals’ hormonal reaction to extended heat exposure and the significant reduction in feed intake (Habeeb et al. 1997). To reduce metabolic heat generation, thyroid hormones drop in response to animals’ exposure to heat stress conditions (Abdalla et al. 1991). The high ambient temperatures have an impact on hormone concentrations that are available for cellular metabolic activities and cellular growth (Habeeb et al. 1992). Many scientists believe that there is a positive relationship between the blood level of thyroid hormones and the rate of animal growth. Thyroxin is also thought to be necessary for the cellular metabolism of the mammary gland, which may be important for milk production (EL-Masry and Habeeb 1989). The growth-promoting properties of the thyroxin hormone also have a substantial impact on the protein, fat, carbohydrate, and mineral metabolisms (Habeeb et al. 1992). Extreme heat stress reduces an animal’s ability to release hormone-releasing factors from its hypothalamus, which lowers the release of thyroid hormones, thyroid-stimulating hormone, and pituitary hormones (Habeeb 2020d). Additionally, thyroid depression in cattle may result from the thyroid’s interaction with the adrenaline and noradrenaline released in response to elevated temperatures (Christopherson et al. 1978). In addition, an animal experiencing heat stress will have decreased thyroid activity as a result of its body responding to its environment (Silanikove 2000). An animal can survive the stress brought on by a hot environment thanks to the physiological changes brought on by cortisol release. After 20 min of acute heat stress exposure, cortisol levels may be increased and plateau in 2 h (Christison and Johnson 1972).

The hyperglycemic action of glucocorticoid hormones, which promote gluconeogenesis and result in the expected increase in glucose use in heat-stressed animals, may explain the rise in cortisol levels during acute heat stress (Marai and Habeeb 1998). The catabolic hormone cortisol, which is present at higher levels in heat-stressed animals, is another factor that stress-related effects on the adrenal gland may potentially influence. Among the hormones known to play a critical role in body thermoregulation are thyroxin, cortisol, insulin, and aldosterone. There is substantial debate in the literature about how hot weather affects cattle’s plasma cortisol levels, according to Marai and Habeeb (2010). In various trials, heat stress boosted plasma glucocorticoids, while in others, it either had no impact or declined sharply. The catabolic hormone cortisol, which is present at higher levels in heat-stressed animals, is another factor that stress-related effects on the adrenal gland may potentially influence. Among the hormones known to play a critical role in body thermoregulation are thyroxin, cortisol, insulin, and aldosterone. There is substantial debate in the literature about how hot weather affects cattle’s plasma cortisol levels, according to Marai and Habeeb (2010). In several trials, heat stress boosted plasma glucocorticoids, while in others, it either had no impact or declined sharply.

### Blood glucose level

Exposure to high ambient temperatures is connected to decreased glucose levels in cattle (Shwartz et al. 2009; Brien et al. 2010). According to Shaffer et al. (1981), the rapid use of blood glucose by the respiratory muscles due to the increase in respiration rate in hot climates led to a drop in blood glucose content during heat stress. The rise in temperature was followed by a significant drop in blood glucose levels. This drop in glucose level during heat exposure is to be largely due to the decrease in insulin and thyroxin concentrations, which is strongly associated with the reduction in energy uptake. Other potential explanations for the decrease in glucose include the significant dilution of blood and bodily fluids brought on by an increase in water intake in hot weather and an increase in glucose utilization to provide more energy required for high respiratory activity (Habeeb et al. 1992). According to Webster (1976), decreased glucose consumption, a suppressed release of both catabolic and anabolic enzymes, and a decreased metabolic rate all contribute to increased glucose concentration under conditions of heat stress. Glycogenolysis and gluconeogenesis are increased with a commensurate decrease or increase in blood glucose levels, respectively, in accordance with Habeeb et al. (1992).

### Blood protein and fat profile

In comparison to mild temperatures, total protein and albumin concentrations decreased in hot environments. According to Habeeb et al. (2011), a variety of factors, including the reduction in feed nitrogen intake that occurs under heat stress conditions and the reduction in protein synthesis as a result of the depression of anabolic hormonal secretion like T_4_ or T_3_ and insulin, may contribute to the decrease in serum protein and its fractions. When exposed to the severe heat of the summer, the buffalo calves’ blood total lipid and total cholesterol concentrations drastically fell by 9.9 and 9.3%, respectively. The decrease in blood biochemical component concentrations that occurred as a result of subjecting the animals may have been caused by the elevation in body water content (Habeeb et al. 1992). Since acetate serves as the main building block for the synthesis of cholesterol, the drop in acetate concentration that occurs after being exposed to heat stress may also be the cause of the drop in cholesterol concentration (Marai and Habeeb 1998). Because blood components dilute under heat stress conditions, total lipid and cholesterol levels may have decreased. A drop in feed consumption or an increase in water consumption may be to blame for this dilution. From another point of view, the winter season had much lower levels of total protein, albumin, globulin, glucose, urea, and creatinine concentrations, as well as AST and ALT activity, than the levels in the hot summer season (Gaafar et al. 2021).Crossbred cows (Brown Swiss x Baladi) in the thermal neutral group had considerably higher levels of total protein, cholesterol, triglycerides, glucose, and thyroid hormones than those in the hot climate group (Teama 2016). Animals under heat stress had greater urea-N concentrations (Habeeb et al. 2012; El-Masry et al. 2010; Shwartz et al. 2009). The improvement in rumen nitrogen balance may be the cause of the increase in urea nitrogen concentration following heat stress exposure (Erasmus et al. 1992). Additionally, an increase in muscle breakdown may be the cause of the spike in urea-N in heat-stressed animals (Kamiya et al. 2006). High levels of urea in heat-stressed animals may be caused by a low energy-to-protein ratio and gluconeogenesis or the breakdown of proteins when there is insufficient energy for growth (Momtmurro et al. 1995).

### Blood enzyme activities and mineral concentration

During the humid summer, farm animals’ aspartate aminotransferase and alanine aminotransferase liver enzyme activities drastically rise (Habeeb et al. 2012; El-Masry et al. 2010). Since blood enzymes and metabolism are closely connected, Marai and Habeeb (1998) showed that external environmental factors, including feeding procedures, the kind of shelter, and various other aspects of animal management, frequently and easily change blood enzyme levels. The study by Wankar et al. (2014) revealed significantly increased aspartate aminotransferase and alanine aminotransferase during thermal stress.

Blood mineral concentrations in the blood of animals significantly decrease during heat stress exposure. The decrease in aldosterone and parathormone hormone secretions, which is connected to an increase in urinary mineral excretion on the one hand and an increase in body fluid and water turnover rates, which help wash out these minerals during heat stress, may be the cause of the decreased blood minerals in heat-stressed animals (Habeeb et al. 1992). In addition to fluids, the body needs electrolytes, which are chemical ions that balance the body’s fluids both within and outside of cells. All physiological measurements were considerably higher in the summer than they were in the winter for all physiological measurements (Gaafar et al. 2021).

This disruption impacts enzymatic responses, such as an increase in transaminase enzyme activity, cortisol level, and declines in insulin, T_4_, T_3_, and aldosterone levels (Habeeb et al. 2018c). Most blood metabolites fall as a result of these disruptions. The result of these abnormalities is a reduction in growth, milk production, and reproductive capacity (Habeeb et al. 2000). Particularly when farm animals are exposed to high external temperatures, the outer warm air receptors in their bodies transmit inhibitory nerve impulses to the hypothalamus area (appetite center) that controls their hunger, causing the animals to consume less food and lowering their thermal load. Several factors, such as the consumption of dry matter, reproduction, and milk production, are negatively impacted by high environmental temperatures (Habeeb et al. 2018b). Consequently, less substrate is available for the synthesis of hormones and the generation of heat. Extreme heat also reduces the level of hormone-releasing factors from the hypothalamic center, which restricts pituitary hormonal release and, as a result, reduces thyroid hormone secretion. Because of these changes, which have an effect on reproduction and productivity, the average daily increase, total body weight, and weaning weight of the animals were all lower in the summer than in the winter (Gaafar et al. 2021). The hypothalamus obtains nerve impulses from peripheral and core receptors that are stimulated by high environmental temperatures. The hypothalamus then activates its protective evaporative and non-evaporative cooling systems to avoid the increase in body temperature (Habeeb 2020e). The animal will die from heat stroke if none of these defense mechanisms is able to avoid the increase in animal body temperature (Pereira et al. 2008). Continued exposure of the animals to high environmental exposure inhibits the release of hormone-releasing factors from the hypothalamic center, which in turn results in a decrease in the levels of hormones. Milk production, growth, and reproduction are all harmfully affected because enzymatic activities are impeded by a decrease in substrate and hormonal levels as presented in Fig. 1 (Kamal 1975).

## The negative effect of high environmental temperatures on growth parameters of ruminants

### Growth and daily body gain

Growth is controlled by well-proportioned nutrients, hormones, and enzymes that have an impact on both heredity and the environment. Extreme climate stress in tropical and subtropical locations is predictable to obligate a deleterious influence on animal condition and productivity by affecting a variety of biological and biochemical changes (Habeeb et al. 1989). When temperate zone animal breeds are exposed to the high environmental temperature atmosphere, their efficiency is reduced by roughly 50%, and these changes in biological processes affect both male and female animals’ growth (Habeeb 2020c). When compared to times of moderate weather, the average daily body gain of buffalo calves is considerably reduced during periods of acute high temperature stress, according to a study by Habeeb et al. (2007). The latter authors found that buffalo calves underwent significant decreases in daily body gain when exposed to high ambient temperatures of 36.0 and 32.0 °C as compared to 18.0 and 22.6 °C, respectively. Habeeb et al. (2009) noted individual variances in the body weight gain that decreased as a result of the calves being subjected to demanding summertime conditions. Habeeb et al. (2012) reported that the high environmental circumstances significantly reduced the daily body gain of buffalo calves by 18.1, 17.41, and 8.65%, respectively, in the first, second, and third months of the hot summer season. Atta et al. (2014) found also that daily body gain values of bovine calves were considerably lower in the hot summer than in the mild climate of winter, with decrease values of 55.2, 60.2, and 57.4% in the first, second, and third months of the hot summer season, respectively. Habeeb et al. (2014) discovered that the hot summer season caused a highly significant reduction in the body weight gain of bovine calves by 30 kg through 3 months at a rate of 333.9 g daily, and the drop in percentage exceeds 45% as compared with the winter’s moderate climate conditions. A highly significant decrease in daily body gain (DBG) and daily dry matter intake (DMI) in the calves due to higher THI was detected. The amount of deterioration in DBG increases with increasing THI. DBG decreased significantly by 18.6% at THI of 74.9 (spring season) and by 41.1% at THI of 85.5 during the summer season compared to THI of 68.1 (winter season). Moreover, DBG decreased significantly by 27.6% at THI of 85.5 during the summer season compared to THI of 74.9 during the spring season. The degree of decline in daily DMI also increases with increasing THI values. Daily DMI decreased significantly by 9.05% at THI 74.9 (spring season) and by 21.4% at THI 85.5 during the summer season compared to THI 68.1 (winter season). DBG decreased significantly by 13.5% at THI 85.5 during the summer season, when compared to DBG at THI 74.9 during the spring season. DMI was decreased daily by 36.0 g and 85 g during the spring and summer seasons, respectively, compared to winter. These results designate that the extreme deteriorating effect of heat stress on both DBG and daily DMI was detected in the summer season (Habeeb et al. 2022).

The effectiveness of conversion of DMI to DBG ratio was improved significantly by 13.1% at THI 74.9 during the spring season and by 33.6% at THI 85.5 during the summer season compared to those in winter 68.1. With increasing THI, the depression in DBG was more than the decline in daily DMI; therefore, food conversion (DMI/DBG) increases when the value of THI increases (Habeeb et al. 2022).

### Total and daily solids gain

Total body solid (live body weight minus total body water) values were shown to significantly decrease in Friesian cows from winter (126.5 kg) or spring (118.0 kg) to summer (91.0 kg), as presented by Kamal and Seif (1969). Total body solid levels were 100 times lower in growing buffaloes at 32 °C than at 18 °C (Kamal et al. 1972). According to Kamal et al. (1978), buffalo total body solids decrease from spring (110.9 kg) to summer (59.5 kg) and from summer when solar radiation is abundant (58.6 kg). Average body solid content decreased by 16% as ambient temperature increased in the climate control room, and Holstein and Friesian calves responded equally to heat stress (Kamal 1982). Average body solid content decreased by 16% as ambient temperature increased in the climate control room, and Holstein and Friesian calves responded equally to heat stress (Kamal 1982). Kamal and Habeeb (1999) found that high temperatures resulted in a significant decrease in total body solids in both male and female Friesian calves.

When compared to the winter season, the stressful summer heat significantly decreased the solid daily gain (total body solids/days of experiment) of bovine calving by 8.0 kg over 3 months at a rate of 88.4 g daily, with the percentage decrease reaching more than 33%. When comparing the two breeds, the results revealed that crossbred calves (Egyptian bovine cows × Brown Swiss bull) had a total body weight of 20.4 kg more than purebred calves, with a daily body gain of 226.1 g. Similar to this pattern, crossbred calves gained 91.6 g of solids per day and 8.2 kg more overall than purebred calves (Habeeb et al. 2014). Average daily body gain of purebred and crossbred native calves was, respectively, 35.9% and 30.4% lower in the hot summer than it was in the mild climate of the winter season (Habeeb et al. 2014). Regarding the impact of the crossing method on daily body gain, the growth characteristics of the crossbred calves were better than those of the purebred calves. These results are explained by the discovery that the enhanced growth performance in crossbreds is caused by heterosis in the offspring’s growth rate (Habeeb et al. 2002). According to Nasr et al. (1997), native cows crossed with Friesian or Brown Swiss bulls had the highest live body weights at birth and weaning. This superiority was mostly due to heterosis in the offspring’s growth rate. Another study discovered that crossing Boer goats with Spanish, Nubian, or Angora goats increased birth weight, weaning weight, and average daily body gain (Brown and Machen 1997). Crossbreeding, according to El-Fouly et al. (1998), significantly increases the body weight gain of calves. This superiority was related to heterosis in the offspring’s growth rate. The latter authors came to the conclusion that Brown Swiss bulls and Native cows were crossbred successfully to increase body weight at birth, weaning, and at 12 months of age. Similar authors also believed that this crossbreeding was successful in strengthening low-producing native cattle. Similar results were also obtained in cattle, sheep, and goats by Rodriguez et al. (2011), Ahuya et al. (2002), and Haque et al. (2011).

The high growth rates were likely brought on by heterotic and additive gene effects for growth and adaptation qualities in cooperation, according to Norris et al. (2002), who found that all of the crosses displayed greater daily body weight increase values than purebred animals. Habeeb et al. (2002) noted, in this regard, that crossbred calves (native cow × Brown Swiss bull) have a genotype that is preferable to that of purebred calves (native cows × native bull) because crossbred calves have a nice structure and type of genes that gathered in a pure Brown Swiss bull and were transferred into local calves. El-Fouly et al. (1998) found that crossbred calves (native cows × Brown Swiss bull) had better food metabolism and absorption than native calves and needed less energy to maintain their body weight. As far as adaptation goes, Molee et al. (2011) found that mixed-breed Holsteins perform better than purebred Holsteins and are also more tolerant of heat stress. Habeeb et al. (2014) found that in winter and summer, respectively, crossbred calves (native cow × Brown Swiss bull) gained 243.0 and 102.0 g and 195.0 and 83.0 g more daily body gain and solid daily gain than purebred calves, demonstrating that crossbred calves are superior in daily body gain and solid daily gain to purebred calves under mild winter climatic conditions and hot summer climatic conditions. The later authors concluded that increased temperature has an adverse effect on growth performance by increasing tissue catabolism and decreasing anabolic activity.

The drop in voluntary feed intake of vital nutrients, particularly metabolizable energy for both weight maintenance and weight gain, is the main cause of the loss in anabolism. Under conditions of heat stress, this results in a reduction in assembly per unit of food (Morrison and Lofgreen 1979). Most of the increase in tissue catabolism is localized in fat depots and/or lean body mass (Habeeb et al. 1992). Higher levels of catecholamines and glucocorticoids, for example, are associated with lower levels of body amino-N and endogenous DNA and RNA purine catabolism (EI-Fouly and Kamal 1979). Young animals’ nitrogen balance declines dramatically in hot weather, although not as dramatically as adult animals’, which have negative nitrogen balance. This phenomenon may be brought on by the inability of young animals’ well-known fast rate of protein synthesis to entirely counteract heat-induced protein catabolism (Habeeb et al. 1992).

The hypothalamus region that regulates hunger receives inhibitory nerve signals from the peripheral thermal receptors when the ambient temperature is high. As a result, the amount of feed or dry matter consumed decreases, as do the substrates for enzyme activity, hormone synthesis, and heat production (Kamal 1982). Furthermore, animals in conditions of acute heat stress have hormone-releasing factors blocked, which results in lower pituitary hormonal output and decreased anabolic hormone release (Habeeb et al. 1989). High relative humidity and ambient temperatures may be harmful to an animal’s health due to dehydration, tissue catabolism, and a lack of metabolic energy for growth. This is because breathing becomes more frequent in warmer weather, which requires more energy (Habeeb et al. 1992). In heat-stressed animals, the rise in glucocorticoids and catecholamines, the rise in insulin levels, the fall in T_4_ and T_3_ levels, the decline in feed intake and digestibility, and the reduction in growth characteristics and increase in tissue destruction indicated by total body solids losses may all be contributing factors (Bernabucci et al. 1999). Animals under heat stress limit their food intake to try to produce less metabolic heat since ruminants’ feeding heat rise, in particular, makes up a great percentage of the body’s overall heat output (Kadzere et al. 2002). Enzymatic activity is being slowed, hormone levels are dropping, and the body temperature is rising; the metabolism is slowed as a result, which prevents daily weight gain. A decrease in thyroid stimulating hormone, an increase in glucocorticoid hormone, or a combination of these variables may also contribute to the summertime drop in thyroid hormone levels. Additionally, the thyroid may interact with the thermoregulatory hormones adrenaline and noradrenaline, resulting in a reduction in weight growth in both live and solid gains (Habeeb et al. 2020a).

## The negative effect of high environmental temperatures on milk yield and composition of ruminant

For milk production, ruminants need upper critical temperatures of between 24 and 30 °C. When temperature averages rose by 3.2, 8.8, and 1.6 °C above normal for dairy cattle, their daily milk yield fell by 4.5, 6.8, and 14%, respectively, and when temperature averages decreased by 7 °C below normal, their daily milk yield improved by 6.5% (Petkov 1971). In early, mid, and late lactation, Bober et al. (1980) found that milk yield was reduced by 25.0, 41.0, and 47.0%, respectively, after 72 h from the initiation of heat exposure. In contrast to the low-producing animals, which lost just 0.65 kg per day at 30 °C, the high-producing cows presented an average loss of 2.0 kg per day (Vanjonack and Johnson 1975). According to Kamal et al. (1989), the average milk output of Friesian cows is 30% lower in a hot environment (38 °C) than in a cool environment (18 °C). Rodriguez et al. (1985) discovered that in Friesian cows, the proportions of protein and fat in milk declined between 8 and 37 °C. Habeeb et al. (2000) found that the average weekly milk production of buffaloes during the hot summer months was significantly lower than that obtained from buffaloes during the winter months in all lactation numbers. Buffaloes exposed to high ambient temperatures had a 16.6% decrease in milk production, as determined by the overall average of the six lactations. Summertime milk total solids, butterfat, protein, and lactose levels were much lower than wintertime levels. According to economic analysis, six buffaloes were subjected to the sweltering heat of the Egyptian summer, which led to a poor milk production reduction of 51.4 kg and a milk total solids loss of 11 kg in their milk. Due to the elevated ambient temperature and the temperature-humidity index, West (2003) found that hyperthermia has a considerable effect on milk components as well as dry matter intake. Friesian cows housed at 38 °C generated on average less total solids, fat, protein, ash, and lactose than when the same animals were kept at thermoneutral environmental temperatures, with declines of 28.0, 27.0, 7.0, 22.7, and 30.0%, respectively, according to research by Habeeb et al. (1989).

Milk output losses seem to be favorably correlated with cow milk yield. The sensitivity of cattle to thermal stress increases with an increase in milk supply, and the threshold temperature at which milk losses occur decreases. The investigations by Nardone et al. (2010) found that keeping high-milk-producing cattle in warmer climates accelerated metabolic heat generation, which in turn caused an increase in respiratory rate and a drop in milk output. In the tropics and subtropics, Molee et al. (2011) discovered that Holsteins mixed with indigenous breeds performed better and were more heat stress tolerant than pure-bred Holsteins. According to Purwanto et al. (1990), non-lactating cows with lower milk yields (18.5 kg/day) or greater yields (31.6 kg/day) produced 27 and 48% more heat while having a lower body weight than non-lactating cows (752, 624, and 597 kg for non-lactating, low, and high producers, respectively). The stage of lactation plays a significant role in how dairy cows react to heat. When compared to their early and late lactating counterparts, Johnson et al. (1998) found that dairy cows in the middle of their lactation were the most susceptible to heat. In fact, when exposed to heat, dairy cows that were mid-lactating showed a greater reduction in milk output (38%) than other animals. The magnitude of the milk supply reduction in Murrah buffaloes ranged from 10 to 30% in the first lactation to 2 to 20% in the second or third lactation, according to Upadhaya et al. (2007), but it was less severe at the mid-lactation stage than at the late or early stage. Cows are under enough heat stress to diminish milk production and fertility when their rectal temperatures are more than 39.0 °C and their respiration rates are more than 60 respiratory per minute (Kadokawa et al. 2012).

Due to the fact that lactating cows consume more feed and produce more heat than non-lactating heifers, lactating cows are more susceptible to heat stress (Berman 2005). The risk of heat stress increases for cows when the outside temperature is higher than their body temperature, which can result in a 50% reduction in milk supply (Ben-Salem and Bouraoui 2009). According to Reyad et al. (2016), milk production and Temperature-Humidity Index (THI) have a bad association. The fact is that the elevated THI exposed animals to heat stress, which in turn decreased milk supply. The maximum milk output was discovered when the THI was lowest, and the lowest milk yield was discovered when the THI was highest. The highest percentages (%) of total solids, solids-not-fat, fat, protein, lactose, and ash content in milk were found in October, whereas the lowest percentages (%) were found in July due to the high THI value. These results demonstrate that in Bangladesh, heat stress has a considerable effect on milk production and milk content in Holstein-Friesian crossbred cows. The amount of milk produced decreased by 21% when the daily THI value climbed from 68 to 78, whereas dry matter intake increased by 9.6%, according to Bouraoui et al. (2002). Every point in the THI readings above 69 resulted in a 0.41 kg per cow per day decrease in milk output. Ozrenk and Inci’s (2008) study found that the proportions of milk fat, protein, and total solids in cow milk were highest in the winter and lowest in the summer. Compared to late-lactating or low-yielding cows, early nursing and high-yielding cows often have more immediate and severe impacts. When compared to thermoneutral settings, the amount of milk produced and the proportion of milk components such as fat, protein, total solids, and solids not-fat were among the characteristics that dramatically decreased during heat stress (Teama 2016).

The reduction in food intake has been identified as a key factor restricting milk production since it has been connected to a condition of negative energy balance (Wheelock et al. 2010). The main reaction to heat exposure is a decrease in feed consumption, which results in a reduction in milk yield and milk components in dairy cattle (Habeeb 2020b). The hypothalamus region that regulates hunger receives inhibitory nerve impulses from peripheral heat receptors when the ambient temperature is high in the summer. By decreasing feed intake, the animals’ heat load is decreased. As a result, substrates for enzyme activity, hormone synthesis, and heat production become less available (Habeeb 2022). Lack of energy, substrates, and hormonal levels may be the cause of the decrease in milk yield and components in heat-stressed lactating cows. Furthermore, the elevated levels of cortisol seen in such animals may be related with the drop in milk production and quality observed in animals exposed to high ambient temperatures (Habeeb 2020c).

Due to higher maintenance expenses, which were predicted to rise by 20%, ruminants used less energy efficiently to produce milk when environmental temperatures in a hot region reached 35 °C. Additionally, ruminants utilize digestible energy at a 35.4 °C less efficiently than they would in an environment with a temperature of 18 °C (West 1993). The reduced synthesis of hormone-releasing factors by the hypothalamus region causes the metabolic pathways to slow down. The deficiency of energy, substrates, hormones, and enzymes has the effect of severely impairing protein consumption. In these conditions, protein catabolism outpaces protein synthesis, resulting in a negative nitrogen balance. The breakdown of protein tissues is a result of elevated glucocorticoid hormone levels, which are protein catabolic. The rise in glucocorticoid hormones could be explained by an increase in gluconeogenesis, which converts amino acids in proteins to their corresponding α-keto acids (Alvarez and Johnson 1973). Increased catecholamine or decreased insulin, both of which are required for protein synthesis, may have an effect on tissue degeneration (Habeeb 2020a). In addition, ruminants exposed to high ambient temperatures experience disruptions in their metabolism of minerals, vitamins, lipids, and carbohydrates. As a result, there is an imbalance between the minerals and nitrogen, which lowers the creation of heat, protein synthesis, and the availability of the minerals required for milk biosynthesis. The drop in the levels of other hormones, including thyroxin and insulin, may also be connected to the reduction in milk production and alteration in milk composition in heat-stressed animals (Habeeb et al. 2018a, 2018b).

## The negative effect of high environmental temperatures on reproductive efficiency of ruminant

High environmental temperatures are one of the major problems that affect farm animals’ potential to breed virtually everywhere in the world. Increased temperatures and humidity impair feed consumption and hormone levels, which in turn impair dairy cattle’s capacity to reproduce. High ambient temperatures can weaken the immune system, injure an embryo in development, and lead to problems with reproduction like poor semen quality and smaller babies (Habeeb 2020b).

### Female

High ambient temperatures may also affect rates of conception and pregnancy (Gantner et al. 2011). Two of the main causes of poor reproductive performance are low estrous detection and fetal or embryonic losses. In the postpartum period, the typical interval between consecutive inseminations can increase to about 40–50 days because only about half of all bouts of estrus are identified. Profitability and reproductive effectiveness suffer as a result. High environmental temperatures significantly reduce ruminant conception and pregnancy rates, and the decrease accelerates when the extreme environmental temperature on the day of artificial insemination exceeds 30 °C (Stevenson et al. 1983). Conception rates ranged from 40 to 50% to less than 10% in the months with the highest average temperatures and less than 10% in the months with the lowest average temperatures (Badinga et al. 1985). Lactating animals commonly struggle to maintain a normal body temperature in heat-stressing settings due to the high rates of internal heat production associated with lactation, which affects conception rates in lactating animals more than in heifers (Wolfenson et al. 1995). Heat stress can affect the appearance of estrous behavior, ovarian follicular growth, oocyte competence, and embryonic development, all of which can lead to disruption of the reproductive process (Mondal et al. 2017). Heat stress causes an increase in endometrial prostaglandin release, which leads to both early regression of the corpus luteum and embryo loss. Heat stress reduces behavioral estrus intensity and duration and has detrimental effects on embryonic mortality; as a result, fewer animals are observable in estrus in heat stress environments (Beatty et al. 2006). The summer had a longer interval between conception and parturition than the winter did (López-Gatius et al. 2006). The intrauterine environment is altered in heat-stressed cows, resulting in decreased blood supply to the uterus and an elevation in uterine temperature. These modifications restrict embryonic development, increase the frequency of early embryo death, and decrease the percentage of successful inseminations (Rivera and Hansen 2001). The rates of conception and pregnancy in animals living in subtropical conditions decreased from 31.6% and 26.3% to 11.5% and 9.9%, respectively, whereas the rate of embryonic loss increased from 11.5% at the lower THI to 22.2% at the higher THI (El-Tarabany and EL-Tarabany 2015). The rates of abortion and stillbirth increased from 3.6% and 3.8% at low THI to 7.2% and 5.9% at high THI. The rate of fetal loss increased from 17.1% at low THI to 24.9% at higher THI. The elevation in the THI was the cause of each of these outcomes. Reduced production of gonadotropin-releasing hormone and luteinizing hormone caused by heat stress prevents ovulation and the expression of estrus activity (Temple et al. 2015). Cows under heat stress also experienced shortened estrus times (Hales et al. 1996). Additionally, heat-stressed female cows are less likely to experience horizontal estrus and frequently display these symbols in the evening, when it is cooler but when they are less likely to be detected (Habeeb et al. 2018b). Heat stress reduces motor activity and other signs of estrus while increasing the likelihood of anestrus and silent ovulation (Singh et al. 2011).

The quality of the oocyte and the embryo’s growth are all impacted by heat stress, along with the release of a number of reproductive hormones. Additionally, it lessens estrus expression. Embryo transfer, which prevents the effects of heat stress on the oocyte and early embryo, is one of the greatest ways to lessen the severity of heat stress (Kadokawa et al. 2012).

Thornton et al. (2015) found that high environmental temperatures increased respiration rate, rectal temperature, oxidative stress, maintenance energy, and acidosis risk, somatic cell count in milk, mastitis health issues, mortality rate, as well as visible and invisible signs of heat stress in livestock. These findings have implications for how well livestock reproduce under climate change. High ambient temperatures also diminish rumen balance and function, rumen rumination, feed intake, conception rate, and milk output while lowering immunity.

Gwazdauskas et al. (1973) found that for every 0.5 °C above the usual range of 38.3–38.6 °C in the uterus, the rate of conception was reduced by 6.9–12.8. Summertime dairy cow conception rates are much lower than those of other seasons in various parts of the world (Flamenbaum and Galon 2010). After in vitro fertilization, summer Holstein cow oocytes were less capable of developing into the blastocyst stage than winter cow oocytes (Gendelman et al. 2010). Poor oocyte quality is linked to lower fertility in repeat-breeder Holstein cows, and heat stress exacerbates this unfavorable effect (Ferreira et al. 2011). High temperature causes differences in follicular growth by raising the number of small and medium follicles and lowering the dominant follicle’s capacity to utilize dominance (Wolfenson et al. 1995). Numerous tissues of the female reproductive tract, including the follicle, oocyte, and embryo, are affected by heat stress in expressions of cellular activity. Additionally, it increases the secretion of follicle-stimulating hormone and decreases inhibit circulation levels (Hansen 2009). Estrus detection is more difficult under conditions of heat stress because dairy cows show fewer symptoms and have shorter estrus durations in the summer than they do in the winter. According to Sakatani et al. (2012), the walking activity of Japanese Black cattle during estrus decreased in the summer compared to the winter, and the summer’s estrous cycle lasted longer (23.4 days to 21.5 days). In dairy cows, diminished estrous behavior and a prolonged estrous cycle in the summer may result from pulsatile LH secretion, which is required to induce estradiol-17b generation, and a drop in estradiol-17b concentrations. Delay in ovulation is one effect of the diminished LH pre-ovulatory surge (Siddiqui et al. 2010).

### Male

The most pronounced effects of hyperthermia on male animal reproductive functions include reduced sperm generation, both in quantity and quality, and decreased fertility. During the hot season, *B. indices* bulls in Africa produced less sperm quality (ejaculate volume, sperm concentration, total sperm number, and percentage of normal sperm cells). In comparison to buffalo raised under temperate environmental circumstances, subtropical summers are hot and result in a decrease in scrotal circumference, testicular consistency, tone, size, and weight in the same breeds of animals (Chauhan and Ghosh 2014).

It can be concluded that ruminants raised during the hot summer months are typically found in tropical and subtropical regions, where they are exposed to extremely high environmental temperatures for almost 6 months of the year. These conditions encourage abrupt changes in their biological processes, which ultimately reduce their capacity for reproduction and production. Therefore, management measures are necessary to lessen the effects of temperature stress circumstances, especially during the hot summer season, in order to achieve maximum performance in the hot environment. Different methods for minimizing heat stress in animals include modifying the environment, lowering the animal’s heat output, and assisting the animal in dispersing the heat load and reversing the negative consequences by physical, physiological, and dietary methods.

## Data Availability

All authors confirmed that the availability of data and materials support their published claims and comply with field standards. All authors affirmed that the information and resources available support their stated claims and adhere to industry standards. Each author attests that the bespoke or available software applications support their claimed claims and adhere to industry standards.
